# Prevalence and factors associated with self-reported sexually transmitted infections in Myanmar: analysis of national survey data

**DOI:** 10.3389/fpubh.2026.1954535

**Published:** 2026-09-15

**Authors:** Myo Tint, Phyo Wai Thaw, Napaphat Poprom, Aksara Thongprachum

**Affiliations:** 1 Faculty of Public Health, Chiang Mai University, Chiang Mai, Thailand; 2 Graduate of the Master of Public Health (Global Health) Program, Faculty of Public Health, Thammasat University, Bangkok, Thailand

**Keywords:** factors associated with, HIV knowledge level, Myanmar demographic and health survey, prevalence, self-reported sexually transmitted infections, sexual health

## Abstract

**Introduction:**

Sexually transmitted infections (STIs) continue to pose a significant public health issue worldwide, especially in low- and middle-income countries (LMICs). However, nationally representative evidence on the prevalence and factors associated with self-reported STIs (SR-STIs) in Myanmar remains limited. This study aimed to estimate the prevalence of SR-STIs, identify factors associated with SR-STIs, and examine differences in STI risks among sexually experienced women and men in Myanmar.

**Method:**

This study analyzed secondary data from the 2015–16 Myanmar Demographic and Health Survey. A total of 8,693 women and 3,196 men aged 15–49 years who had ever had sexual intercourse were included in the analysis. Survey-weighted descriptive statistics, cross-tabulations, and survey-weighted logistic regression analyses were conducted separately for women and men to identify factors associated with SR-STIs.

**Result:**

The prevalence of SR-STIs was higher among women than men (8.0% vs. 6.7%). Among women, medium and higher HIV knowledge level, barriers to healthcare access, and current contraceptive use were significantly associated with increased odds of SR-STIs. Women residing in coastal, delta, and central zones, as well as those in the richer wealth quintile, had lower odds of SR-STIs compared with their counterparts. Among men, sexual debut after 18 years was associated with lower odds of SR-STIs, while married men had lower odds compared with single men.

**Discussion:**

The findings provide nationally representative evidence that may inform gender-sensitive STI prevention strategies, efforts to improve access to sexual and reproductive healthcare services, and the integration of STI prevention into HIV and family planning programs. Given the age of the data, updated STI surveillance and population-based studies are needed to assess the current burden of STIs and guide contemporary prevention and control strategies in Myanmar. Nevertheless, these findings provide an empirical foundation for understanding the burden of SR-STIs and its associated factors for informing future research and public health planning.

## 1 Introduction

Sexually transmitted infections (STIs) are caused by viruses, bacteria, fungi, or parasites that are primarily transmitted through sexual contact (1). They can be spread through vaginal, oral, or anal intercourse (1, 2) and may also be transmitted from mother to child during pregnancy, childbirth, or breastfeeding, as well as through exposure to infected blood or blood products (2). The global burden of STIs on health and socioeconomic development remains insufficiently recognized as a major public health concern (3). According to the World Health Organization (WHO), more than one million curable STIs are acquired every day worldwide among individuals aged 15–49 years, with the majority of infections having no symptoms. In 2020 alone, an estimated 374 million new infections were reported among people in this age group, involving one of the four major curable STIs: chlamydia, gonorrhea, syphilis, and trichomoniasis (4). More than 30 pathogens can be transmitted through sexual contact, and individuals may experience multiple infections simultaneously (5). Because many infections remain unnoticed, untreated STIs can lead to serious long-term health complications, including cancer, chronic pelvic pain, ectopic pregnancy, and infertility, while also increasing the risk of other STIs (5, 6). In addition, STIs and human immunodeficiency virus (HIV) commonly coexist because they share similar modes of transmission, while socio-behavioral factors further intensify their combined burden, particularly among vulnerable populations (7). Both ulcerative and non-ulcerative STIs significantly increase infectiousness and susceptibility to HIV infection due to biological mechanisms such as mucosal disruption and inflammatory responses that facilitate viral transmission (8). Despite the substantial global burden of STIs, prevention and treatment responses remain inadequate in many countries (9). Strengthening integrated HIV and STI prevention services, particularly for high-risk and vulnerable populations in regions with high STI prevalence, is essential to improve sexual and reproductive health outcomes and achieve global health targets (9).

STIs also impose a substantial health and economic burden, particularly in developing countries (4, 10). Approximately 75–85% of the annual new cases of curable STIs occur in these nations, and STIs contribute to 17% of economic losses associated with poor health (10). Owing to their high prevalence, especially in developing countries, STIs contribute considerably to productivity losses among individuals and communities (3). In these settings, untreated STIs represent one of the leading contributors to disability-adjusted life years lost among women of reproductive age, surpassed only by maternal conditions and HIV (3). Despite this, STI control in developing countries remains challenging due to low policy priority, limited resources, weak screening and treatment services, and insufficient sexual health education. Persistent poverty, stigma, and discrimination against vulnerable populations further contribute to continued STI transmission and poor disease control (10).

Myanmar has made significant progress in tackling the HIV epidemic across all age groups over the past two decades (2005–2025). New HIV infections among all age groups declined from 0.35 per 1,000 uninfected population in 2005 to 0.15 per 1,000 in 2025, while AIDS-related mortality decreased from 0.32 to less than 0.1 per 1,000 population over the same period. Additionally, an estimated 95% of people receiving antiretroviral therapy (ART) achieved viral suppression in 2025 (11). However, the burden of STIs remains a significant but under-researched public health issue in Myanmar. Although no specific statistics on STIs were available to our knowledge, it was mentioned that the prevalence of Syphilis and other STIs is still high in Myanmar (12). Existing research on Myanmar predominantly focuses on challenges in seeking HIV/STI information and care among young key populations, including female sex workers, men who have sex with men (13), STI prevalence among male highway coach drivers (14), and knowledge, attitude, and preventive behaviors related to HIV/AIDS and STIs among Myanmar migrant populations (15) in specific locations. Additionally, there is a multi-country comparative study that examines associated factors and prevalence of STIs among reproductive-age women in Southeast Asia (16). However, there is relatively limited information regarding the prevalence of STIs across the country and their associated factors specifically for sexually experienced men and women within the general population in Myanmar.

Previous studies using Demographic and Health Survey (DHS) data from low- and middle-income countries have identified multiple factors associated with STI risk, including demographic and socioeconomic status (16–24). In addition, sexual behaviors (16, 18, 20–24), sexual health knowledge, and healthcare service access (16, 18, 20, 21, 23, 24) were identified as significant associated factors with self-reported (SR) STIs. Understanding these factors is critical for informing targeted sexual health interventions, improving access to STI prevention and treatment services, and strengthening national HIV prevention strategies. Furthermore, gender differences also showed different STI risks (17, 18), highlighting the importance of examining men and women separately in epidemiological analyses. Addressing these gaps is essential for designing gender-sensitive interventions and achieving equitable health outcomes.

Therefore, this study aims to estimate the prevalence of SR-STIs and identify their associated factors among sexually experienced men and women in Myanmar using data from the 2015–16 Myanmar Demographic and Health Survey (MDHS). By providing nationally representative evidence, this study seeks to contribute to addressing an important gap in the country’s epidemiological evidence on STIs.

## 2 Methods

### 2.1 Data source

This study used secondary data from the 2015–16 MDHS, using the Individual Women’s Recode (IR) and Men’s Recode (MR) datasets (25). The MDHS was a nationally representative survey conducted by the Ministry of Health and Sports in Myanmar in collaboration with ICF International (ICF) (25). The 2015–16 MDHS applied a stratified two-stage sampling design, in which 442 clusters (123 urban and 319 rural) were selected from approximately 4,000 primary sampling units (PSUs), followed by the systematic selection of 30 households per cluster, yielding 13,260 households (25). The original survey interviewed 12,885 women and 4,737 men aged 15–49 years between December 2015 and July 2016 (25). Detailed information on the original survey’s sampling procedures, questionnaire administration, interviewer training, and data collection is available in the MDHS report (25). The present study did not involve primary data collection, interviews, or questionnaire administration.

### 2.2 Type of study

This study employed a cross-sectional study design based on secondary analysis of data from the 2015–16 MDHS to examine the prevalence of SR-STIs and their associated factors among sexually experienced women and men aged 15–49 years.

### 2.3 Sample derivation

The study included sexually experienced women and men aged 15–49 years who reported ever having sexual intercourse and had complete information on the outcome variable. The age range of 15–49 years was consistent with the MDHS target population and the standard DHS framework. Respondents with missing data or “do not know” responses were excluded from the analysis. The final analytical sample consisted of 8,693 women and 3,196 men (Figure 1).

**Figure 1:**
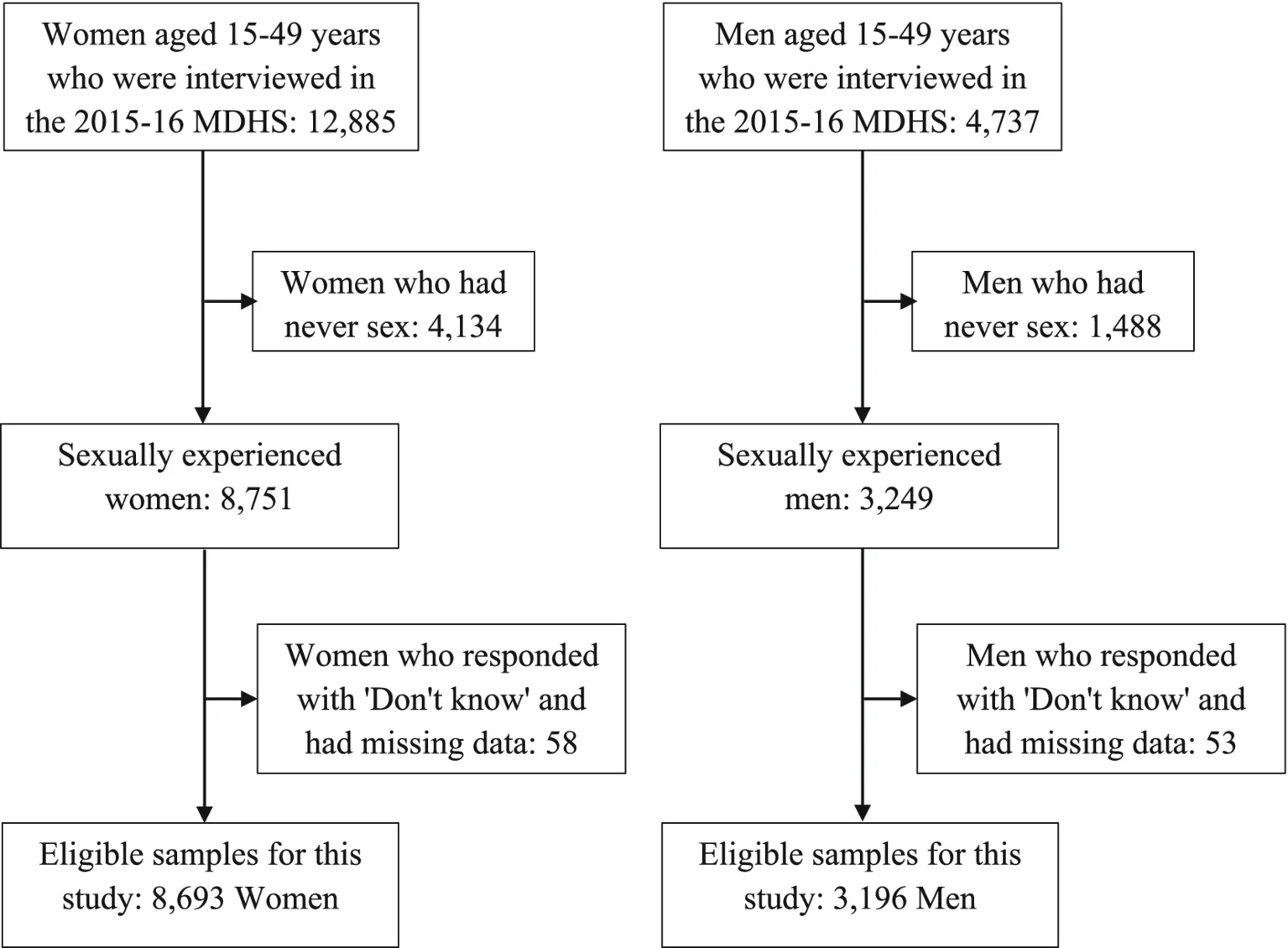
Sampling flowchart.

### 2.4 Variable measurements

#### 2.4.1 Outcome variable

The outcome variable was self-reported sexually transmitted infections (SR-STIs). Following DHS guidelines (26), respondents were classified as having STI symptoms “Yes = 1” if they reported experiencing at least one of the following conditions during the 12 months preceding the survey: an STI or STI symptom, abnormal (or bad-smelling) genital discharge, or a genital sore/ulcer. Respondents reporting none of these conditions were categorized as “No = 0”. SR-STI indicators from DHS datasets have been widely used as proxy measures for STI morbidity in existing studies (16–18, 22, 27–30).

#### 2.4.2 Explanatory variables

Variable selection was informed by evidence from prior research and the biopsychosocial (BPS) model, originally proposed by George L. Engel in 1977, which views health and disease as products of interacting physiological, psychological, and sociocultural factors (31). Applied to SR-STIs, the framework provided a basis for considering individual, behavioral, and social/structural factors associated with SR-STIs. Based on this theoretical concept, the explanatory variables were grouped into three domains: health knowledge and service utilization factors, sexual behavior factors, and sociodemographic factors. Health knowledge and service utilization factors included HIV knowledge level, awareness of other STIs, barriers to healthcare access among women, and ever having HIV testing. Adapted from a previous study (32), HIV knowledge level was measured using six questions related to HIV transmission and prevention: (1) reduce risk of getting HIV: always use condoms during sex, (2) reduce risk of getting HIV: have 1 sex partner only, who has no other partners, (3) a healthy looking person can have HIV, (4) can get HIV from mosquito bites, (5) can get HIV by sharing food with person who has aids, (6) can get HIV by witchcraft or supernatural means. One point was assigned for each correct response, resulting in a total score ranging from 0 to 6. Consistent with previous studies, respondents were categorized as having low (scores 0–3), medium (score 4), and high HIV knowledge (scores 5–6). Awareness of other STIs was assessed using three questions: (1) ever heard of STI, (2) ever heard of other STIs, (3) ever heard of AIDS, categorized as having awareness (if the response was “Yes” to any one question) or no awareness (if the response was “No” to all questions) (33). Barriers to healthcare access among women were assessed based on difficulties in getting permission for medical help, getting money for treatment, distance to a health facility, and reluctance to seek medical help alone. Women reporting “Yes” to any question were categorized as “Have at least one big problem”, and those reporting “No” to all questions were categorized as “Have no big problem” (34–36). Ever having HIV testing was categorized as “Yes” or “No.”

Sexual behavior factors included age at sexual debut (both women and men), current contraceptive use among women, circumcision status among men, condom use at last sexual intercourse among men, and exchange of money for sex among men. Age at sexual debut was categorized into ≤18 years and >18 years, and current contraceptive use among women was categorized as using or not using contraceptive methods (16). The remaining variables in this category: circumcision status, condom use at last sex, and exchange money for sex, were coded “Yes” or “No”, similar to the original dataset.

Sociodemographic factors included age group, place of residence, geographical zone, educational attainment, wealth quintile, marital status, working status, and media exposure. Consistent with previous studies, age group was categorized into 15–24 years and 25–49 years (16), marital status was grouped into single, married, and formerly (widowed, divorced, or separated) (19), and Myanmar states and regions were grouped into four geographical zones: hilly, coastal, delta, and central zones (37). Media exposure was assessed using three variables indicating the frequency of exposure to media, including reading newspapers or magazines, listening to the radio, and watching television. Each variable was originally categorized as “not at all,” “less than once a week,” and “at least once a week.” A composite media exposure variable was then constructed by categorizing respondents as having exposure if they reported exposure to at least one of the three media sources (either less than once a week or at least once a week). Respondents who reported no exposure (not at all) to all three media sources were categorized as having no exposure (20). Residence (urban, rural), education level (no education, primary, secondary, higher), wealth quintile (poorest, poorer, middle, richer, richest), and working status (not working, working) were classified according to the dataset’s original coding.

### 2.5 Data management and statistical analysis

Data management and statistical analyses were performed using Stata version 15.1. Sampling weights (v005/1,000,000 and mv005/1,000,000) were applied to account for unequal probabilities of selection and non-response. The complex survey design, including clustering and stratification, was accounted for using the “svyset” command.

Separate analyses were conducted for men and women because some explanatory variables were sex-specific and to examine differences in STI risks between the two sexes. Descriptive statistics were used to summarize participants’ characteristics and SR-STI prevalence. Frequencies and weighted percentages were reported. Missing data were handled using a variable-specific available-case approach for descriptive analyses. This approach minimized unnecessary exclusion of otherwise eligible participants with available information for other variables. Then, survey-weighted cross-tabulations were performed to examine the distribution of SR-STIs across explanatory variables.

Given the binary outcome and the complex sampling design of the MDHS, unadjusted logistic regression models and multivariate survey-weighted logistic regression models were conducted separately by sex to estimate the associations between explanatory variables and SR-STIs. The survey-weighted models accounted for sampling weights, clustering, and stratification to obtain population-representative estimates and appropriate variance estimates. Unadjusted logistic regression models were first fitted to estimate crude odds ratios (CORs) with 95% confidence intervals (CIs). Multicollinearity among explanatory variables considered for the multivariable models was assessed using variance inflation factors (VIFs), with values below 5 indicating no evidence of substantial multicollinearity. The maximum VIF was 3.26 among women and 3.64 among men. Subsequently, multivariable logistic regression models were fitted to estimate adjusted odds ratios (AORs) with 95% CIs, accounting for potential confounding among the explanatory variables. Variables with no variation in the estimation sample were automatically omitted by Stata. Potential effect modification was assessed by introducing theoretically relevant interaction terms and jointly testing their statistical significance using adjusted Wald tests. Statistical significance was defined as a two-sided *p*-value <0.05.

## 3 Results

### 3.1 Prevalence of SR-STIs and characteristics of study participants by sex

A total of 8,693 women and 3,196 men aged 15–49 years were included in the analysis. The prevalence of SR-STIs was 8.0% among women and 6.7% among men, indicating a higher prevalence among women.

Regarding health knowledge and service utilization factors, high HIV knowledge levels were observed among 41.8% of women and 46.2% of men, while awareness of STIs was reported by 91.7% of women and 93.3% of men. Nearly half of women (49.8%) reported at least one major barrier to healthcare access. HIV testing uptake remained relatively low in both sexes, with only 25.7% of women and 27.0% of men reporting ever having undergone HIV testing.

For sexual behavior factors, most respondents reported sexual debut after 18 years of age (women: 65.7%; men: 80.9%). Among women, 47.4% were currently using contraceptive methods. Among men, only 3.8% reported being circumcised, 3.3% reported condom use at last sexual intercourse, and 2.9% reported ever exchanging money for sex.

Regarding sociodemographic characteristics, the majority of respondents were aged 25–49 years (women: 86.7%; men: 89.2%), about 73.4% of women and 72.5% of men resided in rural areas, and 90% of women and 90.5% of men were currently married. The largest proportion of participants resided in the delta zone for both women (37.2%) and men (38.6%). Primary education was the most common educational level among women (47.2%) and men (41.3%). Wealth quintiles were relatively evenly distributed (around 20%) across both sexes. Most men (95.7%) and approximately two-thirds of women (65.0%) were currently working. Media exposure was high overall, particularly among men (91.2%) compared with women (84.6%). See Table 1.

**Table 1:** Prevalence of SR-STIs and characteristics of study participants by sex variables.

Variables	Women (*n* = 8,693)		Men (*n* = 3,196)	
	No.	Weighted %	No.	Weighted %
Outcome variables				
Self-reported STI infection				
No	7,926	92.0%	2,965	93.3%
Yes	767	8.0%	231	6.7%
Independent variables				
Health knowledge and service utilization factors				
HIV knowledge level	(*n* = 7,909)		(*n* = 2,994)	
Low	3,190	39.6%	1,037	34.2%
Medium	1,442	18.6%	600	19.6%
High	3,277	41.8%	1,357	46.2%
Awareness of STI				
Have no awareness	750	8.3%	191	6.7%
Have awareness	7,943	91.7%	3,005	93.3%
Barriers to healthcare access	(*n* = 8,692)			
Have no big problem	4,151	50.2%	NA	NA
Have at least one big problem	4,541	49.8%	NA	NA
Ever had HIV testing	(*n* = 8,691)			
No	6,268	74.3%	2,348	73.0%
Yes	2,423	25.7%	848	27.0%
Sexual behavior factors				
Age at sexual debut	(*n* = 8,337)		(*n* = 2,831)	
≤ 18	2,896	34.3%	573	19.1%
> 18	5,441	65.7%	2,258	80.9%
Current contraceptive use				
Not using	4,836	52.6%	NA	NA
Using	3,857	47.4%	NA	NA
Circumcised			(*n* = 3,184)	
No	NA	NA	3,047	96.2%
Yes	NA	NA	137	3.8%
Condom use at last sex			(*n* = 2,856)	
No	NA	NA	2,756	96.7%
Yes	NA	NA	100	3.3%
Exchange money for sex				
No	NA	NA	3,093	97.1%
Yes	NA	NA	103	2.9%
Sociodemographic factors				
Age group				
15–24 years	1,178	13.3%	347	10.8%
25–49 years	7,515	86.7%	2,849	89.2%
Residence				
Urban	2,324	26.6%	867	27.5%
Rural	6,369	73.4%	2,329	72.5%
Geographical zone				
Hilly	2,723	18.6%	990	18.7%
Coastal	1,612	11.9%	493	9.7%
Delta	1940	37.2%	772	38.6%
Central	2,418	32.3%	941	33.0%
Education	(*n* = 8,691)			
No education	1,346	15.7%	432	13.7%
Primary	4,005	47.2%	1,284	41.3%
Secondary	2,680	29.3%	1,275	38.5%
Higher	660	7.8%	205	6.5%
Wealth quintile				
Poorest	1865	20.9%	665	20.3%
Poorer	1785	20.4%	663	20.1%
Middle	1766	20.0%	642	20.0%
Richer	1727	19.5%	652	20.1%
Richest	1,550	19.2%	574	19.5%
Marital status				
Single	14	0.1%	204	5.5%
Married	7,816	90.0%	2,873	90.5%
Formerly	863	9.9%	119	4.0%
Working status	(*n* = 8,691)			
Not working	3,273	35.0%	163	4.3%
Working	5,418	65.0%	3,033	95.7%
Media exposure				
Have no exposure	1,358	15.4%	295	8.8%
Have exposure	7,335	84.6%	2,901	91.2%

NA, not applicable because these variables are not available for specific sexes in the original datasets.

The percentages were calculated among respondents with available information for each variable, resulting in variable-specific denominators and frequencies that may not sum to the overall sample of 8,693 (women) and 3,196 (men) because of missing information for some variables.

### 3.2 Distribution of SR-STIs across explanatory variables by sex

Among women, statistically significant associations with SR-STIs were observed for HIV knowledge level (*p* < 0.001), awareness of other STIs (*p* = 0.015), barriers to healthcare access (*p* = 0.003), current contraceptive use (*p* = 0.031), place of residence (*p* = 0.044), geographical zone (*p* = 0.003), education level (*p* = 0.009), and media exposure (*p* = 0.007). Higher prevalence of SR-STIs was observed among women with high HIV knowledge (10.0%), those aware of STIs (8.3%), those reporting at least one major barrier to healthcare access (9.2%), current contraceptive users (8.8%), urban residents (9.6%), women residing in coastal zone (9.4%), women with higher education (10.0%), and those exposed to media (8.4%).

Among men, statistically significant associations with SR-STIs were identified for age at sexual debut (*p* < 0.001), exchange of money for sex (*p* = 0.004), and marital status (*p* < 0.001). Higher prevalence of SR-STIs was observed among men reporting sexual debut at ≤18 years (11.1%), those who had ever exchanged money for sex (15.9%), and single men (20.3%). No significant associations were observed for the remaining explanatory variables among men. See Table 2.

**Table 2:** Distribution of SR-STIs across explanatory variables by sex.

Variables	Women (Weighted)			Men (Weighted)		
	No (%)	Yes (%)	*p*-value	No (%)	Yes (%)	*p*-value
Health knowledge and service utilization factors						
HIV knowledge level			<0.001			0.771
Low	93.7%	6.3%		92.5%	7.5%	
Medium	91.4%	8.6%		93.6%	6.4%	
High	90.0%	10.0%		93.1%	6.9%	
Awareness of other STIs			0.015			0.066
Have no awareness	94.6%	5.4%		97.1%	2.9%	
Have awareness	91.7%	8.3%		93.0%	7.0%	
Barriers to healthcare access			0.003			
Have no big problem	93.2%	6.8%		NA	NA	NA
Have at least one big problem	90.8%	9.2%		NA	NA	NA
Ever had HIV testing			0.220			0.639
No	92.2%	7.8%		93.4%	6.6%	
Yes	91.2%	8.8%		92.9%	7.1%	
Sexual behavior factors						
Age at sexual debut			0.959			<0.001
≤ 18	92.2%	7.8%		88.9%	11.1%	
> 18	92.2%	7.8%		94.4%	5.6%	
Current contraceptive use			0.031			
Not using	92.7%	7.3%		NA	NA	NA
Using	91.2%	8.8%		NA	NA	NA
Circumcised						0.130
No	NA	NA		93.4%	6.6%	
Yes	NA	NA		88.9%	11.1%	
Condom use at last sex						0.104
No	NA	NA		93.9%	6.1%	
Yes	NA	NA		89.2%	10.8%	
Exchange money for sex						0.004
No	NA	NA		93.5%	6.5%	
Yes	NA	NA		84.1%	15.9%	
Sociodemographic factors						
Age group			0.335			0.546
15–24 years	91.1%	8.9%		92.5%	7.5%	
25–49 years	92.1%	7.9%		93.4%	6.6%	
Residence			0.044			0.114
Urban	90.4%	9.6%		91.8%	8.2%	
Rural	92.6%	7.4%		93.9%	6.1%	
Geographical zone			0.003			0.309
Hilly	90.0%	1.0%		92.8%	7.2%	
Coastal	90.6%	9.4%		91.4%	8.6%	
Delta	91.4%	8.6%		94.5%	5.5%	
Central	94.2%	5.8%		92.7%	7.3%	
Education			0.009			0.475
No education	93.8%	6.2%		95.3%	4.7%	
Primary	92.6%	7.4%		92.9%	7.1%	
Secondary	90.5%	9.5%		92.9%	7.1%	
Higher	90.0%	10.0%		93.5%	6.5%	
Wealth quintile			0.151			0.806
Poorest	91.4%	8.6%		93.5%	6.5%	
Poorer	92.4%	7.6%		92.8%	7.2%	
Middle	92.6%	7.4%		93.8%	6.2%	
Richer	93.0%	7.0%		94.1%	5.9%	
Richest	90.4%	9.6%		92.2%	7.8%	
Marital status			0.744			<0.001
Single	89.7%	10.3%		79.7%	20.3%	
Married	92.0%	8.0%		93.9%	6.1%	
Formely	92.0%	8.0%		96.7%	3.3%	
Working status			0.157			0.520
Not working	91.3%	8.7%		91.7%	8.3%	
Working	92.3%	7.7%		93.3%	6.7%	
Media exposure			0.007			0.831
Have no exposure	94.1%	5.9%		93.7%	6.3%	
Have exposure	91.6%	8.4%		93.3%	6.7%	

NA, not applicable because these variables are not available for specific sexes in the original datasets.

### 3.3 Factors associated with SR-STIs among women in Myanmar

The results of the unadjusted logistic regression and the multivariate survey-weighted logistic regression for women are presented in Table 3. The results of the multivariate survey-weighted logistic regression revealed that women with medium and high HIV knowledge levels had significantly higher odds of reporting SR-STIs compared with those with low HIV knowledge (AOR = 1.47, 95% CI: 1.06–2.04, *p* = 0.020 and AOR = 1.67, 95% CI: 1.25–2.23, *p* = 0.001, respectively). Women reporting at least one major barrier to healthcare access were more likely to report SR-STIs than those without barriers (AOR = 1.35, 95% CI: 1.08–1.69, *p* = 0.009). Current contraceptive users were statistically significantly associated with higher odds of SR-STIs (AOR = 1.26, 95% CI: 1.02–1.55, *p* = 0.030). Awareness of STIs was automatically omitted from the multivariable models because of collinearity in Stata.

**Table 3:** Factors associated with SR-STIs among women in Myanmar.

Variables	COR	95% CI	p-value	AOR	95% CI	p-value
Health knowledge and service utilization factors						
HIV knowledge level						
Low	Ref			Ref		
Medium	1.38	(1.02–1.87)	0.035	1.47	(1.06–2.04)	0.020
High	1.64	(1.29–2.08)	<0.001	1.67	(1.25–2.23)	0.001
Awareness of other STIs^a^						
Have no awareness	Ref			-		
Have awareness	1.58	(1.09–2.30)	0.016			
Barriers to healthcare access						
Have no big problem	Ref			Ref		
Have at least one big problem	1.39	(1.12–1.72)	0.003	1.35	(1.08–1.69)	0.009
Ever had HIV testing						
No	Ref			Ref		
Yes	1.14	(0.92–1.41)	0.220	0.91	(0.73–1.12)	0.367
Sexual behavior factors						
Age at sexual debut						
≤ 18	Ref			Ref		
> 18	1.00	(0.83–1.21)	0.958	0.96	(0.78–1.18)	0.710
Current contraceptive use						
Not using	Ref			Ref		
Using	1.22	(1.02–1.46)	0.032	1.26	(1.02–1.55)	0.030
Sociodemographic factors						
Age group						
15–24 years	Ref			Ref		
25–49 years	0.88	(0.67–1.15)	0.335	0.90	(0.67–1.23)	0.519
Residence						
Urban	Ref			Ref		
Rural	0.76	(0.58–0.99)	0.045	0.87	(0.62–1.21)	0.400
Geographical zone						
Hilly	Ref			Ref		
Coastal	0.94	(0.70–1.25)	0.662	0.62	(0.45–0.86)	0.004
Delta	0.84	(0.61–1.17)	0.304	0.58	(0.42–0.81)	0.001
Central	0.56	(0.40–0.77)	<0.001	0.45	(0.32–0.64)	<0.001
Education						
No education	Ref			Ref		
Primary	1.21	(0.89–1.63)	0.209	1.08	(0.74–1.58)	0.688
Secondary	1.58	(1.15–2.18)	0.005	1.29	(0.83–1.99)	0.264
Higher	1.67	(1.07–2.60)	0.023	1.38	(0.75–2.52)	0.301
Wealth quintile						
Poorest	Ref			Ref		
Poorer	0.87	(0.64–1.19)	0.379	0.83	(0.59–1.17)	0.281
Middle	0.85	(0.63–1.14)	0.279	0.76	(0.54–1.06)	0.106
Richer	0.79	(0.56–1.12)	0.190	0.61	(0.42–0.89)	0.010
Richest	1.13	(0.82–1.55)	0.454	0.72	(0.49–1.07)	0.105
Marital status						
Single	Ref			Ref		
Married	0.75	(0.18–3.11)	0.695	0.93	(0.20–4.28)	0.926
Formerly	0.82	(0.19–3.47)	0.783	1.14	(0.24–5.39)	0.870
Working status						
Not working	Ref			Ref		
Working	0.87	(0.72–1.05)	0.157	0.91	(0.74–1.12)	0.392
Media exposure						
Have no exposure	Ref			Ref		
Have exposure	1.46	(1.11–1.92)	0.007	1.32	(0.96–1.82)	0.089

COR, crude odds ratio; AOR, adjusted odds ratio; CI, confidence interval; Ref, reference category. ^a^ Variable omitted from the multivariable model because it had no variation among respondents with nonmissing HIV knowledge information.

Among sociodemographic factors, geographical zone and wealth quintile were statistically significantly associated with SR-STIs after adjustment. Compared with women residing in the hilly zone, those living in the coastal (AOR = 0.62, 95% CI: 0.45–0.86, *p* = 0.004), delta (AOR = 0.58, 95% CI: 0.42–0.81, *p* = 0.001), and central zones (AOR = 0.45, 95% CI: 0.32–0.64, *p* < 0.001) had lower odds of reporting SR-STIs. Women in the richer wealth quintile were less likely to report SR-STIs compared with those in the poorest quintile (AOR = 0.61, 95% CI: 0.42–0.89, *p* = 0.010). Interaction effects between (1) HIV knowledge level and barriers to healthcare access and (2) wealth index and place of residence were also explored to assess potential effect modification. Neither interaction was statistically significant (HIV knowledge × healthcare barriers: adjusted Wald test, *F*(2, 410) = 0.40, *p* = 0.674; wealth index × residence: adjusted Wald test, *F*(4, 408) = 0.93, *p* = 0.444). Therefore, the original main-effects multivariable model was retained.

In the unadjusted analysis, residence, education level, and media exposure were significantly associated with SR-STIs; however, these associations were no longer statistically significant after adjustment for potential confounders. HIV testing history, Age at sexual debut, age group, marital status, and working status were not significantly associated with SR-STIs in either unadjusted or adjusted analyses. See Table 3.

### 3.4 Factors associated with SR-STIs among men in Myanmar

The association between later sexual debut and lower odds of SR-STIs remained statistically significant in the adjusted model (AOR = 0.52, 95% CI: 0.32–0.85, *p* = 0.009). Married men were less likely to report SR-STIs compared with single men (AOR = 0.33, 95% CI: 0.11–0.93, *p* = 0.037). However, the association between exchange of money for sex and SR-STIs was no longer statistically significant after adjustment for potential confounders. No other explanatory variables were significantly associated with SR-STIs in both analyses.

In the unadjusted analysis, men reporting sexual debut after 18 years had lower odds of SR-STIs compared with those reporting sexual debut at ≤18 years (COR = 0.47, 95% CI: 0.31–0.72, *p* = 0.001). Men who had ever exchanged money for sex had significantly higher odds of SR-STIs (COR = 2.74, 95% CI: 1.34–5.60, *p* = 0.006). Marital status was also significantly associated with SR-STIs, with married and formerly married marital status groups reporting lower odds compared with single men. See Table 4.

**Table 4:** Factors associated with SR-STIs among men in Myanmar.

Variables	COR	95% CI	*p*-value	AOR	95% CI	*p*-value
Health knowledge and service utilization factors						
HIV knowledge level						
Low	Ref			Ref		
Medium	0.84	(0.51–1.40)	0.513	0.74	(0.43–1.29)	0.288
High	0.92	(0.60–1.40)	0.700	0.69	(0.43–1.12)	0.138
Awareness of STI^a^						
Have no awareness	Ref			-		
Have awareness	2.48	(0.91–6.78)	0.075			
Ever had HIV testing						
No	Ref			Ref		
Yes	1.09	(0.77–1.53)	0.639	0.90	(0.55–1.48)	0.683
Sexual behavior factors						
Age at sexual debut						
≤ 18	Ref			Ref		
> 18	0.47	(0.31–0.72)	0.001	0.52	(0.32–0.85)	0.009
Circumcised						
No	Ref			Ref		
Yes	1.78	(0.84–3.76)	0.135	1.86	(0.73–4.77)	0.195
Condom use at last sex						
No	Ref			Ref		
Yes	1.86	(0.87–3.98)	0.109	1.16	(0.40–3.32)	0.785
Exchange money for sex						
No	Ref			Ref		
Yes	2.74	(1.34–5.60)	0.006	1.38	(0.52–3.67)	0.521
Sociodemographic variables						
Age group						
15–24 years	Ref			Ref		
25–49 years	0.87	(0.56–1.36)	0.546	1.58	(0.78–3.19)	0.201
Residence						
Urban	Ref			Ref		
Rural	0.73	(0.49–1.08)	0.115	0.78	(0.46–1.32)	0.355
Geographical zone						
Hilly	Ref			Ref		
Coastal	1.21	(0.72–2.04)	0.468	1.19	(0.59–2.45)	0.616
Delta	0.75	(0.43–1.32)	0.323	0.80	(0.40–1.62)	0.549
Central	1.02	(0.62–1.67)	0.941	1.36	(0.73–2.54)	0.327
Education						
No education	Ref			Ref		
Primary	1.56	(0.93–2.62)	0.093	1.33	(0.70–2.52)	0.385
Secondary	1.55	(0.90–2.68)	0.112	1.30	(0.62–2.74)	0.492
Higher	1.41	(0.62–3.17)	0.409	1.34	(0.50–3.62)	0.559
Wealth quintile						
Poorest	Ref			Ref		
Poorer	1.11	(0.66–1.84)	0.700	0.91	(0.50–1.65)	0.756
Middle	0.95	(0.59–1.52)	0.816	0.74	(0.39–1.39)	0.354
Richer	0.90	(0.52–1.56)	0.718	0.74	(0.35–1.56)	0.424
Richest	1.21	(0.70–2.09)	0.487	0.93	(0.42–2.03)	0.847
Marital status						
Single	Ref			Ref		
Married	0.25	(0.16–0.39)	<0.001	0.33	(0.11–0.93)	0.037
Formerly	0.14	(0.04–0.42)	0.001	0.28	(0.05–1.45)	0.129
Working status						
Not working	Ref			Ref		
Working	0.79	(0.38–1.62)	0.520	0.78	(0.27–2.23)	0.645
Media exposure						
Have no exposure	Ref			Ref		
Have exposure	1.07	(0.56–2.03)	0.831	1.37	(0.55–3.40)	0.501

COR, crude odds ratio; AOR, adjusted odds ratio; CI, confidence interval; Ref, reference category. ^a^ Variable omitted from the multivariable model because it had no variation among respondents with nonmissing HIV knowledge information.

## 4 Discussion

This study examined the prevalence and associated factors with SR-STIs among sexually experienced women and men in Myanmar using nationally representative MDHS 2015–16 data. The findings showed that the prevalence of SR-STIs was 8.0% among women and 6.7% among men, with women reporting a higher prevalence than men. The factors associated with SR-STIs also differed between women and men, highlighting the importance of considering sex-specific patterns in understanding STI vulnerability and informing prevention and control strategies in Myanmar.

The higher prevalence of SR-STIs among women compared with men is consistent with findings from previous studies conducted in LMICs (17, 18, 38), and South and Southeast Asian countries (39). This finding also aligns with the fact that women are disproportionately affected by STIs across their lifespan, particularly in developing countries. Owing to biological susceptibility related to female urogenital anatomy, combined with social, cultural, and economic inequalities, women experience a higher burden of STIs and HIV than men (40, 41). In addition, women in LMICs encounter additional challenges in STI prevention due to inadequate healthcare infrastructure, limited availability of sexual health services, and restrictive gender-related cultural attitudes toward sexuality (42). Myanmar women frequently have limited autonomy in negotiating condom use, even when they believe their partners may be involved in sexual relationships outside the partnership (43). Another study also highlighted that women’s inability to negotiate for safer sex in many LMICs puts them at higher risk of getting infected with STIs (44). These findings highlight the importance of adopting gender-sensitive STI prevention and control strategies in Myanmar.

Among women, higher HIV knowledge was associated with increased odds of self-reported STIs, which is inconsistent with findings from Cambodia and the Philippines, where women who had never heard of HIV/AIDS were more likely to report STIs or STI-related symptoms (16). However, a previous study conducted in an LMIC, Papua New Guinea (18), and in Southeast Asian countries, the Philippines (45) and Indonesia (24), reported similar directions of association to this study. Although this association may appear counterintuitive, it is possible that women with comprehensive HIV/AIDS knowledge may also have greater healthcare knowledge, making them more likely to seek healthcare for STI symptoms (46), leading to increased self-reporting. Another possible explanation is that women with previous exposure to sexual health education or healthcare services may also be more likely to identify STI-related symptoms accurately (45). These findings suggest that knowledge alone may not necessarily translate into safer sexual behaviors or reduced STI risk. Therefore, sexual health education programs in Myanmar may consider not only improving knowledge but also strengthening behavioral change interventions and access to preventive services.

This study also found that women who reported at least one big problem in seeking healthcare services had higher odds of SR-STIs. This could be explained by the finding that social and structural barriers increase women’s vulnerability to STIs by limiting their access to essential sexual and reproductive healthcare services. Financial and social disadvantages further restrict women’s access to HIV/STI prevention and treatment services, contributing to poorer sexual health outcomes (41). In Myanmar, gender disparities in healthcare accessibility, particularly in rural and underserved settings (47) may further hinder timely access to sexual and reproductive healthcare services. This finding highlights the importance of improving equitable access to free, affordable and accessible STI screening and treatment services for women, particularly those facing socioeconomic or geographic disadvantages.

Current contraceptive use among women was associated with higher odds of SR-STIs. This finding is consistent with previous DHS-based studies (16, 17). One possible explanation is that many contraceptive methods used by women, such as oral contraceptives, injectables, or intrauterine devices, do not protect against STIs (4). Women relying on non-barrier contraceptive methods may therefore engage in sexual activity without condom use, increasing their vulnerability to STIs (48). In addition, contraceptive users may have greater exposure to reproductive health services, resulting in increased awareness and reporting of STI symptoms. These findings reinforce the need to integrate STI prevention messages, including condom promotion and safer-sex counseling, into family planning services.

Geographical disparities in SR-STIs were also observed among women. Women residing in coastal, delta, and central zones had lower odds of SR-STIs than those living in hilly regions. Myanmar is an ethnically diverse country comprising predominantly Burman-majority regions and ethnic states inhabited by indigenous people, many of whom live in hilly, remote areas. These regions experience substantial health disparities, including sexual and reproductive healthcare services, due to limited healthcare infrastructure, inadequate transportation, and reduced access to healthcare services (49). Such barriers to healthcare access may contribute to the higher prevalence of SR-STIs observed in hilly regions of Myanmar. The findings underscore the necessity of implementing geographically targeted interventions that are culturally acceptable and tailored to the specific needs of populations, particularly in underserved and hard-to-reach regions.

Women from the richer wealth quintile were less likely to report SR-STIs than those from the poorest quintile. This finding is consistent with a previous study (24). Recent research, however, has shown mixed findings, with some studies reporting contradictory results (50, 51) and others revealing no significant associations (17, 19, 22). A possible reason for this observation could be that higher socioeconomic status enhances access to healthcare services, STI prevention information, and health-promoting behaviors. Wealthier women may often have greater autonomy to seek timely care, whereas economically disadvantaged women face barriers such as healthcare affordability, limited health literacy, and increased vulnerability to risky sexual relationships. This finding emphasizes the importance of addressing socioeconomic inequalities in STI prevention programs.

Among men, later sexual debut was associated with lower odds of SR-STIs. This finding is similar to previous studies conducted in the Philippines (45), and another LMIC, Ghana (23). Supporting evidence from other Asian countries, including China (52) and Nepal (53) have also demonstrated that early sexual debut is associated with an increased risk of STI/HIV acquisition and engagement in sexual risk behaviors. A possible explanation may be the greater involvement of adolescents in risky sexual behaviors, such as multiple sexual partnerships, sexual experimentation, inconsistent condom use, and alcohol use. These behaviors during the transition to adulthood can increase the susceptibility of sexually active men to various STIs (23, 54, 55). Young people in Myanmar have limited sexual and reproductive health knowledge, with 38% of adolescents unaware that pregnancy can result from a single sexual encounter and only 16.67% of individuals aged 15–24 years having accurate knowledge of HIV prevention (56). Those initiating sexual activity at younger ages with limited sexual health knowledge may therefore be more vulnerable to STI acquisition. This finding highlights the importance of comprehensive sexuality education programs aimed at delaying sexual initiation and promoting safer sexual behaviors among adolescents and young adults.

Married men had lower odds of SR-STIs compared with single men, similar to a previous study (55) that showed a significant association in an unadjusted model. Nevertheless, a previous study conducted in South and Southeast countries (39) has reported contradictory results, and other studies revealed no significant associations (18, 23). Single men may be more likely to engage in higher-risk sexual behaviors, including multiple sexual partnerships and inconsistent condom use, which is often associated with high STI risk. Married men tend to have more stable sexual relationships and lower exposure to high-risk sexual networks (57) and may possess greater sexual and reproductive health knowledge compared to single individuals.

This study has important public health implications for STI prevention and control in Myanmar. Currently, STI management in Myanmar primarily relies on a syndromic approach because laboratory diagnostic capacity remains limited, with services delivered through government AIDS/STI Prevention and Control Teams and collaborating non-governmental organizations (12). The identified sex-specific associated factors with SR-STIs highlight opportunities to strengthen existing prevention strategies. Integrating comprehensive sexual health education, improving women’s access to healthcare, and incorporating STI prevention into family planning and HIV services may enhance current control efforts. Tailored interventions for economically disadvantaged women and high-risk geographical areas, together with strengthened integrated HIV/STI services, could improve sexual and reproductive health outcomes in Myanmar.

## 5 Strengths and limitations

This study has several important strengths. Using nationally representative 2015–16 MDHS data and applying survey weights with adjustment for the complex sampling design enhanced the representativeness and generalizability of the findings to the Myanmar population. In addition, separate analyses for women and men enabled the identification of sex-specific associated factors with SR-STIs, providing more targeted public health insights. The study also examined a broad range of factors, including healthcare access barriers among women and geographical differences across Myanmar, which remain relatively underexplored in STI research in the country. Therefore, the findings contribute to the empirical evidence base on SR-STIs in Myanmar and may provide useful context for future research and public health planning related to gender-sensitive and context-specific STI prevention.

However, some limitations should also be considered when interpreting the findings. First, the cross-sectional nature of the survey data prevents establishing causal relationships between explanatory variables and SR-STIs. Second, SR-STIs were based on self-reported symptoms rather than laboratory-confirmed diagnoses, which may introduce recall bias, reporting bias, and potential misclassification. Because many STIs are asymptomatic, the prevalence of SR-STIs may have been underestimated. Additionally, some potentially relevant behavioral and healthcare-related variables were collected only for one sex in the MDHS, limiting the ability to examine comparable determinants across women and men. Finally, although the 2015–16 MDHS provides the most recent nationally representative data for Myanmar, the study findings should be interpreted with caution, as they may not fully capture the country’s current context following major social, political, and economic changes. Updated nationally representative surveys and strengthened STI surveillance systems are recommended to provide timely evidence for policy and program planning.

## 6 Conclusion

This study demonstrated that SR-STIs remain an important public health concern among sexually experienced women and men in Myanmar, with women reporting a higher prevalence than men. The findings identified distinct gender-specific determinants of SR-STIs, including HIV knowledge level, barriers to healthcare access, contraceptive use, geographical zone, and wealth status among women, and age at sexual debut and marital status among men. These results highlight the complex influence of behavioral, socioeconomic, and healthcare access factors on STI risk. Strengthening integrated and gender-sensitive STI prevention and control strategies, including improved access to sexual and reproductive healthcare services, comprehensive sexual health education, and targeted interventions for vulnerable populations, may help reduce the burden of STIs in Myanmar.

## Data Availability

Publicly available datasets were analyzed in this study. This data can be found at: https://dhsprogram.com/data/new-user-registration.cfm.
